# The Evolution of Offspring Size: A Metabolic Scaling Perspective

**DOI:** 10.1093/icb/icac076

**Published:** 2022-06-03

**Authors:** Amanda K Pettersen, Lukas Schuster, Neil B Metcalfe

**Affiliations:** School of Life and Environmental Sciences, University of Sydney, Sydney, NSW 2006, Australia; Institute of Biodiversity, Animal Health and Comparative Medicine, University of Glasgow, Glasgow G20 0TH, UK; School of Biological Sciences, Monash University, Melbourne, VIC 3800, Australia; Institute of Biodiversity, Animal Health and Comparative Medicine, University of Glasgow, Glasgow G20 0TH, UK

## Abstract

Size at the start of life reflects the initial per offspring parental investment—including both the embryo and the nutrients supplied to it. Initial offspring size can vary substantially, both within and among species. Within species, increasing offspring size can enhance growth, reproduction, competitive ability, and reduce susceptibility to predation and starvation later in life, that can ultimately increase fitness. Previous work has suggested that the fitness benefits of larger offspring size may be driven by energy expenditure during development—or how offspring metabolic rate scales with offspring size. Despite the importance of early-life energy expenditure in shaping later life fitness trajectories, consideration of among-species scaling of metabolic rate at the time of birth as a potential source of general metabolic scaling patterns has been overlooked by theory. Here, we review the patterns and processes of energy expenditure at the start of life when mortality is often greatest. We compile existing data on metabolic rate and offspring size for 191 ectotherm species spanning eight phyla and use phylogenetically controlled methods to quantify among-species scaling patterns. Across a 10^9^-fold mass range, we find that offspring metabolic rate scales hypometrically with size, with an overall scaling exponent of 0.66. This exponent varies across ontogenetic stage and feeding activity, but is consistently hypometric, including across environmental temperatures. Despite differences in parental investment, life history and habitat, large-offspring species use relatively less energy as a proportion of size, compared with small-offspring species. Greater residual energy can be used to fuel the next stages of life, particularly in low-resource environments. Based on available evidence, we conclude that, while large knowledge gaps remain, the evolution of offspring size is likely shaped by context-dependent selection acting on correlated traits, including metabolic rates maintaining hypometric scaling, which operates within broader physical constraints.

## Introduction: energy expenditure at the start of life

**Fig. 1:**
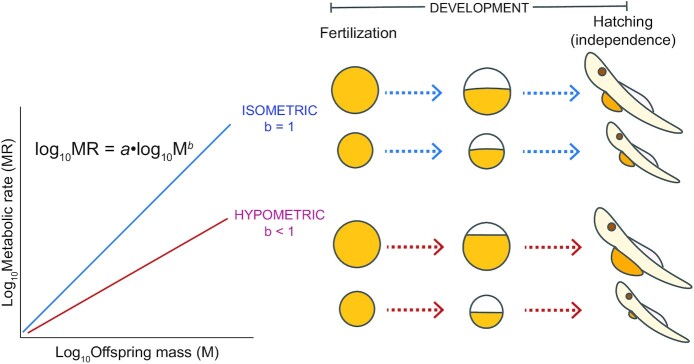
Variation in energy expenditure under isometric versus hypometric scaling with offspring size. Life-history theory assumes that energy expenditure during early life, and therefore the return on parental energy investment, is directly proportional to offspring size, visualized here as an isometric relationship (blue line). Under an isometric relationship, large and small offspring use the same proportion of their initial reserves completing development and will hatch with equivalent residual yolk per unit body mass. However, according to metabolic theory, metabolism scales disproportionately with body size, whereby the scaling exponent (shown here as *b*) is <1 (red line). Under such hypometric scaling, large offspring will hatch with a higher proportion of their initial energy reserves compared with small offspring.

Energy is the currency of life, and the rate of energy expenditure (a.k.a. metabolic rate) reflects how an organism expends energy reserves throughout its life history—from embryo to adult—toward essential processes, including development, growth, maintenance, and reproduction (Stearns 1992; Auer et al. 2018). The start of the life history is often a critical barrier for most metazoans, where high mortality rates reduce to survival to reproduction, and therefore influence fitness more than any other life stage (Kamler 1992). Embryonic development from a fertilized cell to a nutritionally independent juvenile can be costly from an energy perspective—with metazoans using up to 60% of their energy reserves to complete development (Fig. 1; Marshall et al. 2020). For offspring developing in eggs or those with no post-partum care, both the condition of the offspring and the environment it experiences early in life, can influence energy expenditure, with the potential to affect fitness and even the performance of subsequent generations (Plaistow et al. 2006; Pettersen et al. 2016). Early-life energy acquisition and expenditure can also impact ecological dynamics, affecting population demography and connectivity, community structure, and biodiversity patterns (Houde and Zastrow 1993; O'Connor et al. 2007; Schuster et al. 2021). Variation in early-life energy expenditure has clear evolutionary and ecological implications, yet key patterns—and the processes underlying them, have been largely overlooked by metabolic theory.

Here, we provide an overview of existing theory and literature regarding offspring size and energy expenditure. We investigate how incorporating energy expenditure during early-life stages may contribute to our understanding of metabolic theory. We use the term “offspring size” to refer to the initial per offspring investment by the parent (predominantly the mother), including both the developing embryo or larva, and its supplied endogenous energy reserves and nutrients, such as yolk. A considerable number of studies have measured both offspring size and metabolic rates as traits, however metabolic scaling with offspring size has yet to be reviewed among species, potentially due to the difficulties with accounting for developmental stage, metabolic level, and parental provisioning (see the section “Limitations of measuring metabolic scaling of offspring size”). Here, we compile data from these studies to investigate the patterns and implications of allometric scaling for evolution of offspring size. Using phylogenetic comparative analysis—specifically phylogenetic mixed models (Hadfield 2010), we quantify patterns of interspecific scaling of metabolic rate with offspring size for 191 ectotherm species, spanning insects to reptiles (see Supplementary Information for details). We then investigate how the scaling exponent changes across ontogenetic stage (embryos versus larvae), temperature, and activity level (non-feeding versus feeding on exogenous food at the time of hatching/laying). Finally, we discuss potential ultimate and proximate causes of offspring size scaling and provide future directions to address key knowledge gaps.

### What is offspring size?

Offspring size is a fascinating trait that reflects both the parental (often maternal) and offspring phenotype. All metazoans start life as a single-celled zygote, yet offspring size also encompasses the materials that will provision an embryo throughout early development, including crucial sources of lipid, protein, micronutrients, hormones, antioxidants, and antibodies (Eising et al. 2006; Williams 2012). Offspring size, often measured as mass, area, or length, in empirical studies, shows remarkable diversity both within and among species (Bernardo 1996; Marshall et al. 2018). Interspecific variation in offspring size scales over 13 orders of magnitude in metazoans, from a 300 ng bivalve egg to a blue whale calf weighing approximately 3000 kg at birth (Ruud 1956; Sprung 1984). Within a single population, among-individual variation in offspring size can be four-fold, with the majority of total variation in egg size due to among-clutch variation (Christians 2002; Marshall and Keough 2007).

### The evolution of offspring size: a life-history perspective

Understanding why and how offspring size variation is maintained is complex, since initial size can pose direct fitness consequences across generations (Einum and Fleming 2000a; Wolf and Wade 2001; Rollinson and Hutchings 2013). Upon reaching maturity, parents (often mothers) allocate their finite reproductive reserves into provisioning offspring to sustain progress through vulnerable life stages and reach nutritional independence, such as a feeding juvenile. This allocation generally results in trade-offs such as between current and future reproductive output by the parents, or fecundity and offspring quality—whereby mothers can produce either many small, poor-performing offspring or fewer large, high-performing offspring (Vance 1973; Smith and Fretwell 1974; Reznick 1985; Stearns 1989). The allocation of finite reproductive resources can also result in bet-hedging to the extent that within-individual variation in offspring size can exceed among-individual variation in offspring size (Parker and Begon 1986; Marshall et al. 2008). Life-history theory explores patterns and trade-offs of reproduction, which may help to inform how selection operates at the level of populations, and thus ultimately how traits related to fitness evolve (Stearns 1989). Despite consensus that energy is the limiting factor driving trade-offs among key biological processes (Stearns 1989), life-history theory has traditionally not accounted for metabolic scaling patterns that may inform its assumptions. For example, the cost of increasing investment per offspring (such as via offspring size) is expected to result in a concomitant decrease in fecundity for the mother—that is, life-history theory predicts a simple linear trade-off between size and number, and implicitly assumes that small and large offspring require the same amount of energy, as a proportion of their size (Smith and Fretwell 1974). Theory has therefore yet to account for empirical evidence that—as with adults—offspring metabolic rate often scales hypometrically with size (Pettersen et al. 2015; Pettersen et al. 2018). We therefore suggest that known metabolic scaling patterns for adult body size, and the proposed mechanisms that underlie them, can be used to refine, and better understand parental trade-offs in energy allocation among offspring.

### Focus and assumptions of metabolic theory

Metabolic scaling relationships that are central to metabolic theory are typically synthesized from the adult life stage (Kleiber 1932; White and Kearney 2014). The scaling of the rate of energy expenditure (i.e., metabolic rate; MR) with body mass (*M*) is generally well described by a power function

$$\begin{equation*}
{\rm MR} = a{M^b},
\end{equation*}$$

where *a* is the scaling coefficient and *b* is the scaling exponent that describes the slope of the relationship (for both the power law relationship and on a log–log scale). The scaling exponent *b* is expected to range between 0 and 1—thus producing a hypometric (sometimes called negative allometric) relationship (Glazier 2018; Harrison 2018). A hypometric relationship infers that—relative to their size—larger organisms uptake, transform, and expend energy at a lower rate, than their smaller counterparts, per unit body mass. Whether a hypometric relationship is consistent across all life stages—and the potential consequences of variation metabolic scaling across ontogeny—remains underexplored. The little evidence that exists so far suggests that the effect of mass on metabolic rate is life-stage dependent, and energy expenditure per unit mass of adults is unlikely to reflect that of offspring (Epp and Lewis Jr. 1980; Giguère et al. 1988; Post and Lee 1996; Sears et al. 2012; Maino and Kearney 2014; Glazier et al. 2015).

### What are the implications of metabolic scaling with offspring size?

The relationship between offspring size and metabolic rate during early life has been largely disregarded by metabolic theory, despite evidence that these traits are under selection (Sinervo et al. 1992; Einum and Fleming 2000b; Wilson et al. 2009; Monro et al. 2010; Marshall and Monro 2013; Pettersen et al. 2016). With increases in initial offspring size, individuals often show higher survival, growth, and reproductive output, and lower susceptibility to starvation and predation later in life (Hutchings 1991; Moran and Emlet 2001; Marshall et al. 2003; Marshall and Keough 2008). One general mechanism that has been proposed to explain the offspring size-performance relationship is the relative metabolic rate of small and large offspring, or metabolic scaling with offspring size (Pettersen et al. 2015). If, similarly to adults, metabolism scales hypometrically with offspring size, (i.e., *b* < 1), then larger offspring should use proportionally less of their energy reserves completing development than smaller offspring. Consequently, for a given per-offspring energy investment, mothers can either produce fewer, larger, more energy efficient offspring, or many small offspring that waste a higher proportion of their allocated reserves completing development. The implications of hypometric metabolic scaling are perhaps most profound during the early life history, particularly when offspring are nonfeeding and completely reliant on energy reserves supplied in the egg (Mousseau and Fox 1998). While higher mass-specific metabolic rates during juvenile or adult life stages may facilitate faster feeding rates (i.e., energy acquisition) (Biro and Stamps 2010), and an overall faster pace-of-life (Pettersen et al. 2020), nonfeeding offspring will deplete their finite energy reserves sooner. Thus, under hypometric scaling, larger offspring may be able to allocate their greater energy reserves toward growth or larger feeding structures, or to tolerate periods of low food availability. Previous work has shown that larger offspring can indeed hatch with a higher proportion of their initial energy reserves, and that they hatch relatively heavier, and in better condition, than smaller offspring (Pettersen et al 2018; Goulden et al. 1987; 2018). Higher residual yolk at the stage of nutritional independence is known to increase post-hatching growth and survival under low food conditions (Troyer 1987; Murakami et al. 1992; Vidal et al. 2002). Whether the benefits of hypometric scaling for large offspring also translate into higher fitness has yet to be directly tested but is likely to be context dependent.

### Does metabolic rate scale with offspring size among species?

**Fig. 2:**
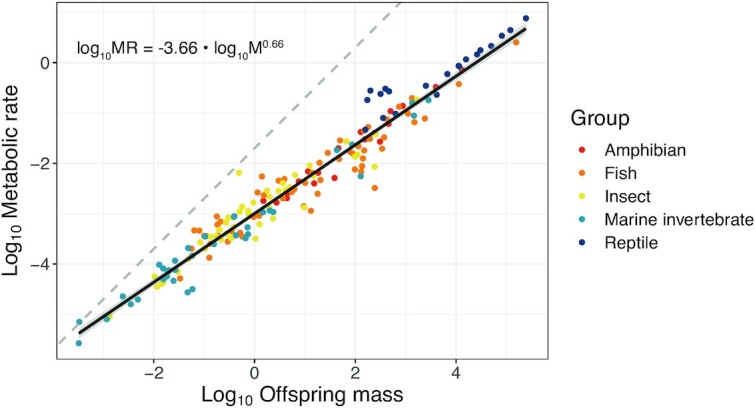
Offspring metabolic scaling with mass for 191 ectotherm species across five groups of taxa (amphibians [*n* = 19], fish [*n* = 64], insects [*n* = 50], marine invertebrates [*n* = 39], and reptiles [*n* = 20]). Log_10_-transformed mass (mg) and metabolic rates (mL O_2_ h^–1^) data adjusted for temperature and phylogeny (see Supplementary Information for details). Dark line shows fitted relationship between log_10_ offspring mass and log_10_ metabolic rate (± standard error), which generates a hypometric scaling exponent *b* of 0.66, significantly different to an isometric relationship (represented by dashed line), but not significantly different to the range of exponents generally predicted for juveniles or adults (c. 0.65–0.80).

We used a literature review to examine the scaling relationship between offspring metabolic rate and size for 191 ectotherm species (see Supplementary Information for details). Among ectotherm species, we found offspring metabolic rate scales hypometrically with offspring mass, with an overall scaling exponent (*b*) of 0.66 (credible interval (CI): 0.56–0.76; Supplementary Table S2; Fig. 2). Our dataset consisted of embryos (*n* = 88) and larvae (*n* = 119) with little or no postnatal care. In our dataset, mass spanned nine orders of magnitude, from blue mussel (*Mytilus edulis*) larvae (3.26×10^–4^ mg; Sprung 1984) to Burmese python (*Pythonmolurus bivittatus*) embryos (2.43×10^5^ mg; Black et al. 1984). Previously, studies have assumed that metabolic mass exponents during early life were similar to those of juveniles and adults (i.e., *b* ∼ 0.8; Winberg 1960; Oikawa and Itazawa 1985; Rombough 1988)—here, we provide evidence that embryos and larvae have similar, if not shallower, scaling exponents than later life stages. Previous studies have also supported higher mass exponents, such as isometric relationships (*b* ∼ 1.0), in larval compared with adult stages of fish (Kamler 1976; Forstner et al. 1983; Giguère et al. 1988; Killen et al. 2007). These differences may be driven by ontogenetic, rather than static intraspecific scaling (Finn et al. 2002; Yagi and Oikawa 2014; Schuster et al. 2019). Our findings suggest that larval stages may show steeper scaling exponents than embryonic stages (*b*_larval_ = 0.72, CI: 0.60, 0.84, *b*_embryonic_ = 0.62, CI: 0.49, 0.76; Supplementary Table S2); however, further investigation within species and clades is warranted.

### What factors influence offspring metabolic scaling?

**Fig. 3:**
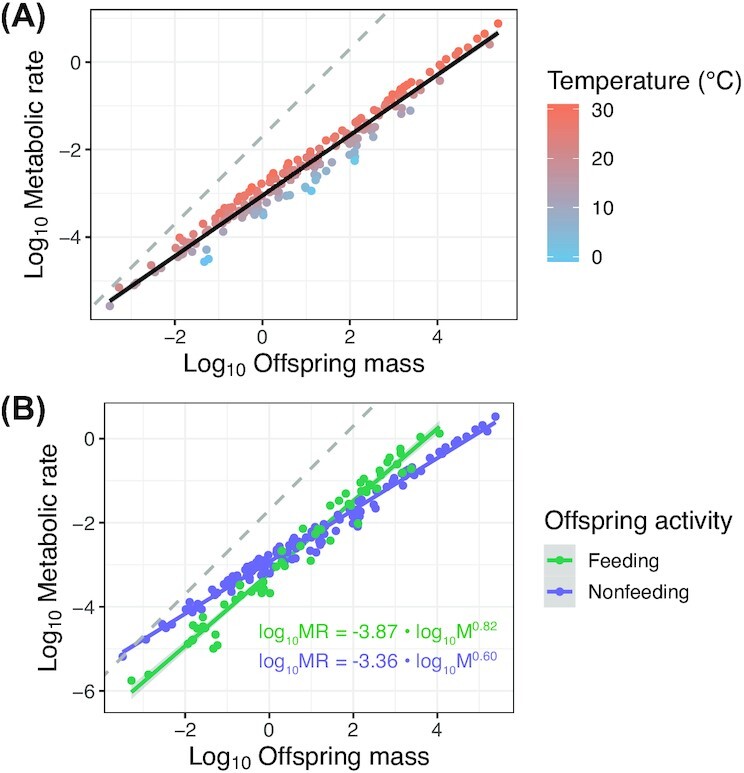
Offspring metabolic scaling relationships among species, with the same data plotted to show the effect of (**A**) environmental temperatures and (**B**) feeding versus nonfeeding activity. Data are adjusted for phylogeny [and temperature in panel (**B**)]. Dashed line represents an isometric relationship (1:1 relationship between log_10_ offspring metabolic rate and log_10_ offspring mass).

The covariance between offspring metabolic rate and mass may depend on extrinsic environmental factors as well as intrinsic characteristics of the organism that vary across ontogeny. Here, we focus on two key factors that are particularly relevant to energy expenditure: developmental temperature and feeding activity. Temperature produces profound effects on physiology, and using the Arrhenius equation, theory predicts that the effect of temperature on metabolic rate (i.e., activation energy) is consistent across body sizes (Gillooly et al. 2001, 2002). The assumption that increases in environmental temperature increase metabolic rate independently of the metabolic scaling relationship has been heavily debated (Glazier 2005). With regards to offspring size, we do not find any evidence for an interactive effect of offspring mass and temperature. Species developing at warmer temperatures have relatively higher metabolic rates (i.e., temperature increases the intercept) than those inhabiting cooler climates; however, there is no significant interaction between mean metabolic rate and mean temperature across species (Fig. 3A). Within species, the trend is less clear. Empirical evidence shows evidence of among-population variation in the thermal sensitivity of key physiological rates (including developmental, growth, and metabolic rates) for the same average body size (Williams et al. 2016; Moffett et al. 2018; Pettersen et al. 2019). Whether offspring mass and temperature interactively affect metabolic rate is still underexplored. The limited available evidence shows that developmental temperature can either have a negative (Laurence 1975; Glazier et al. 2020) or no (Mueller et al. 2011; Pettersen et al. 2019) effect on the scaling exponent. Furthermore, environmental temperatures likely elicit adaptive responses in maternal investment patterns, which may in turn influence the scaling exponent. A body of evidence shows that both within and among species, mothers reared under cool temperatures, or those inhabiting cool climates produce larger offspring than mothers in warm environments; this effect is known as the offspring size–temperature relationship (Yampolsky and Scheiner 1996; Atkinson et al. 2001; Pettersen et al. 2019). One proposed mechanism for this response is that cold temperatures extend development times and are therefore more costly from an energy perspective (Pettersen et al. 2019). Under hypometric scaling, it may be beneficial to produce larger, more energy efficient offspring, to help offset costly development in cool temperatures. Yet, other factors, such as maternal body size (which was not accounted for in our analysis), can constrain the upper limit of offspring size (Lim et al. 2014). Continued investigation of the interplay between hypometric scaling with offspring size and parental investment strategies in response to climatic selection regimes will likely yield important insights.

Intrinsic characteristics of organisms, such as lifestyle have been shown to influence both metabolic rates and scaling coefficients in adult ectotherms (Glazier 2005; Killen et al. 2010). There is also incredible diversity in life histories, and therefore the form and activity level, of offspring (Levin and Bridges 1995). Here, we focus on ectotherm species with no postnatal care, yet within this group, some species produce fully competent young that commence feeding upon or soon after parturition, while others produce offspring that must complete metamorphosis externally to reach a feeding juvenile stage. For offspring developing in eggs, yolk reserves are needed to sustain energy requirements during embryogenesis (Deeming and Ferguson 1991). The form and extent of initial parental investment, often reflected by offspring size, can determine the extent of the non-feeding versus feeding stage and therefore activity level and energy requirements at the start of life (Strathmann 1985). We compared metabolic scaling with offspring size for non-feeding eggs, embryos, and larvae (*n* = 128 species) versus feeding larvae (*n* = 82 species) and find that feeding offspring (0.82, CI: 0.70, 0.96) have a steeper scaling exponent compared with non-feeding offspring (0.60, CI: 0.47, 0.73), however with overlapping credible intervals (Fig. 3B). Non-feeding offspring showed a greater range of offspring mass (3.26×10^–4^–2.43×10^5^ mg) compared with feeding offspring (3.33×10^–4^–1.63×10^4^ mg), driven by mass differences between insect and marine invertebrate larvae compared with vertebrate (reptile and amphibian) embryos. While non-feeding offspring showed overall slightly higher metabolic rates (i.e., intercept), there was no significant difference across groups (see Supplementary Information). A persistent hypometric relationship across offspring feeding activity indicates that there may be similar constraints and/or selection operating to maintain low mass-specific metabolism in large offspring and high mass-specific metabolism in small offspring.

### Why does metabolic rate scale allometrically with offspring size?

Investigating whether selection acts on the covariance between offspring mass and metabolic rate may reveal an ultimate driver of hypometric scaling in early life. There is a growing appreciation of the role of selection in shaping physiological traits, which may be as strong as selection on life history and morphological traits (Strobbe et al. 2010). Recent work proposes that widespread hypometric scaling is shaped by correlational selection, and therefore genetic correlations, between mass and metabolic rate (White et al. 2019; Beaman et al. 2020). Given the evidence that metabolic rate during early life is under selection (see the section “What are the implications of metabolic scaling with offspring size?”), then our observation of hypometric scaling with offspring size may also be shaped by correlational selection for small and large offspring with high and low mass-specific metabolic rates, respectively. A key question that remains is whether offspring hypometric scaling has evolved in response to the same selection pressures as for the adult life stage.

To understand whether macroevolutionary patterns of scaling are driven by microevolutionary processes of selection, measures of selection and heritability within species are needed (Pettersen et al. 2018). Estimates of the heritability of metabolic rate are exceedingly rare, yet a recent summary found the narrow-sense heritability for resting metabolic rate in ectotherms to be 0.19 (±SE: 0.06) (Pettersen et al. 2018). We are only aware of one study measuring heritability of metabolism in eggs, which found that additive genetic effects were small and non-significant in a land snail (Bruning et al. 2013). A recent meta-analysis summarizing selection coefficients (focussed largely on adult stages) found no general trend for selection on metabolic rates (Arnold et al. 2021). The few studies that have measured the relationship between early-life metabolic rate and fitness proxies have found mixed support for selection on trait combinations of either small size and high metabolism and/or large size and low metabolism (i.e., negative correlational selection). For example, field studies show evidence for positive correlational selection (Schuster et al. 2021), negative correlational selection (Bartheld et al. 2015), and no correlational selection (Artacho and Nespolo 2009) between mass and metabolic rates in juvenile stages.

If correlational selection for high metabolic rates in small offspring and low metabolic rates in large offspring is shaping hypometric scaling, then the mechanisms driving this may be due to different strategies adopted by small and large offspring. High metabolic rates can support greater energy output and are often associated with a fast pace-of-life, allowing offspring to complete development and reach a size refuge (thereby escaping predation) sooner, which may be critical in high predation environments (Biro and Stamps 2010). Since smaller offspring also tend to develop faster, there may be correlational selection for fast developing, small offspring when predation or competition during early life is high (Vance 1973; Blanckenhorn 2000; Marshall and Bolton 2007). Conversely, low metabolic rates and low energy allocation, may be favored to reduce energy expenditure, for example, when resources are low (Bochdansky et al. 2005; Burton et al. 2011). In these environments, there may also be selection for mothers to increase their per offspring energy allocation, resulting in large offspring with relatively low metabolic rates per unit mass (Fox et al. 1997; Giesing et al. 2011). Given the lack of empirical data available, it is premature to draw any conclusions regarding selection of offspring size and metabolic rate trait combinations at this stage. However, it seems likely that the evolution of early-life mass and metabolism in natural populations will be context-dependent, and subject to eco-evolutionary feedbacks, such as due to shifts in resource availability, competition, predation, and climate (Nilsson-Örtman et al. 2013; Auer et al. 2018; Auer et al. 2018; Pettersen et al. 2020).

### Potential proximal mechanisms of offspring metabolic scaling

Metabolic theory is dominated by hypotheses exploring physical constraints on resource acquisition and expenditure (West et al. 1997; Glazier 2005; Kooijman 2010). Many of the proximal mechanisms proposed to explain *how* metabolic rate scales hypometrically with adult body size are yet to be applied to offspring size scaling. For example, the exchange or transport of nutrients and waste through distribution networks or across surface boundaries depends on assumptions regarding reserve and structural components, yet offspring composition and structure is insufficiently investigated across the diverse range of developmental modes that exist (Maino and Kearney 2014; Maino et al. 2017). Hence, the data needed to inform parameterization of robust models put forward by metabolic theory is currently lacking.

Oxygen availability is often proposed as a direct constraint to increases in both offspring size and metabolic rate (Einum et al. 2002). With increasing embryo size, the surface area to volume ratio decreases—reducing the efficacy of oxygen transport and setting the upper limits of offspring size (Seymour and White 2006; Rollinson and Rowe 2018). Since high metabolic rates place an increased demand for oxygen, large offspring with high metabolic rates may not survive development, particularly in aquatic environments where oxygen supply can be limited (Rollinson and Rowe 2018). To attain high metabolic rates, offspring may need to have a high surface area:volume ratio—which can primarily be accomplished by reducing offspring size. Despite its intuitive appeal, the oxygen limitation hypothesis may only be relevant for aquatic organisms and therefore not a widespread mechanism for metabolic scaling patterns.

Systematic variation in the composition of different sized offspring may provide a potentially general explanation for hypometric scaling with offspring size. Energy reserves such as yolk contribute to offspring mass yet are metabolically inert. If larger offspring receive a greater amount of yolk relative to their structure, compared with smaller offspring, then a hypometric relationship would be expected. Similarly, increases in offspring size due to higher proportional water content could contribute to mass independently of metabolic rate. So far, data informing how offspring composition scales with size show mixed results. In one study, bryozoan larvae spanning a three-fold size range were found to have similar densities, and therefore presumably proportional yolk reserve (Pettersen et al. 2015), yet for three genera of echinoderms and across five species of killifish, the relationship between egg size and energy content was found to be species-dependent (Moran et al. 2013; Vrtílek et al. 2020). Intraspecific data compiled for over 30 bird species showed that for the majority of (but not all) species, larger eggs have absolutely greater dry masses and energy content, but egg composition varies in direct proportion to changes in egg size (Williams 1994). It appears that the relationship between offspring mass and composition is likely species- and context-dependent; however, further investigation into how offspring composition scales with size and therefore offspring “quality” may help to inform and bridge metabolic and life-history theories.

### Limitations of measuring metabolic scaling of offspring size

There are many logistical and conceptual potential difficulties encountered when comparing mass-metabolic rate relationships (Rombough 1988), which may help to explain the lack of consideration of offspring size in metabolic theory. Here, we discuss five key potential limitations.

1. There are often logistical hurdles to precisely measure the small masses and metabolic rates of embryos and larvae. The measurements of metabolic rates from individuals of varying body mass that are needed to calculate scaling relationships can be difficult to obtain, although recent technological advances are increasingly enabling measurements from insect and marine invertebrate larvae. Nevertheless, 56% (190/341) of studies that met our criteria were published prior to the year 2000—suggesting that data availability has not been a barrier to the synthesis of offspring size scaling.
2. Scaling relationships will greatly depend on whether metabolically inert yolk is included with the mass of the embryo in calculations of metabolic scaling. Embryos can also gain water (e.g., reptile and amphibian eggs; Cunningham and Hurwitz 1936) or lose water (e.g., Rodda 2000), which can influence mass independently of metabolic rate. The decision to measure wet or dry offspring mass, and as either yolk and embryo mass combined or separate, needs to be made clear and justified in the methods, as well as relevant to the specific hypotheses being tested.
3. Scaling relationships can change throughout ontogeny, including across early-life stages (Killen et al. 2007), hence obtaining measures across equivalent stages is challenging (Yagi and Oikawa 2014). Measures of both the initial parental investment as well as metabolic rate and mass (yolk and embryo wet and dry weight) throughout early ontogeny will help account for this variation and improve our understanding of the causes and consequences of metabolic scaling.
4. Metabolic level (i.e., basal, resting, routine, and active) is often accounted for in measures of adult metabolic rates, yet this is difficult to control for in early-life stages, where standard metabolic rates (inactive, post-absorptive state) are often not possible. Our results suggest that feeding offspring species show a steeper scaling exponent than nonfeeding offspring species—whether this is a direct result of feeding activity, or an indirect effect of other unmeasured, correlated traits, is unclear. There may be differences in the allocation of energy between maintenance, development, and post-hatching growth, depending on whether these processes are fuelled by endogenous energy reserves, and/or external resources, that deserve further investigation.
5. There is an extensive range of life histories observed across the metazoan (e.g., fertilization, parental care, developmental modes) and deciding what constitutes an offspring size, for example, embryo mass versus egg content (Mitchell and Seymour 2000), will likely influence among-species comparisons. Nevertheless, development from a single, fertilized cell to a feeding juvenile is ubiquitous, and warrants meeting the challenges associated with defining and categorizing early-life stages. Regardless of the potential pitfalls and limitations, offspring size metabolic scaling relationships may reveal insights into constraints of energy allocation by parents and acquisition by offspring.

Difficulties with attaining precision and controlling for life stage and activity level may have led to traditional metabolic theory overlooking the role of early life in shaping metabolic scaling. Standard or resting metabolic rates are not measurable in developing early-life stages, yet this feature does not justify its exclusion from theory. In contrast, early-life energy expenditure presents a fascinating gap in metabolic ecology that warrants further attention. There is still much insight to be gained about the evolution and maintenance of metabolic hypoallometry by considering energy expenditure from the start of life.

### Future directions

A more holistic approach is needed to identify the causes and consequences of variation in offspring size—one that accounts for both physical constraints on the limits of size, but also the microevolutionary processes that shape the trajectory of offspring size variation. For the development of metabolic life-history theory to inform ecological and evolutionary processes, it is important to capture processes occurring in preadult life stages. This will require accounting for both within-species variation (among individual and population variation, but also ontogenetic scaling) in addition to among-species variation. A central tenet of metabolic theory is to conduct mass-energy balance. To determine how energy is allocated to reserve versus structure, measures of mass and energy reserves at the start and end of the dependent phase are needed and will facilitate further incorporation of the early life history into metabolic theory.

## Supplementary Material

icac076_Supplemental_File

## Data Availability

Presented data and R code for analysis is available on the Open Science Framework: https://osf.io/qrwuz/?view_only=d4fbb221636c487bac52ab680d6ebe18.

## Notes

From the symposium “Causal mechanisms of interspecific metabolic scaling patterns’’ presented at the annual meeting of the Society for Integrative and Comparative Biology virtual annual meeting, January 3–February 28, 2022.
