# Supplementary material for: The Evolution of Offspring Size: A Metabolic Scaling Perspective
Source: Integr Comp Biol. 2022 Jun 3;62(5):1492–502. doi: 10.1093/icb/icac076 (PMC9724151; doi:10.1093/icb/icac076)
Supplement: icac076_Supplemental_File [file icac076_supplemental_file.docx]

**The evolution of offspring size: a metabolic scaling perspective**

**Supplementary Information**

Amanda K. Pettersen^1,2*^, Lukas Schuster^3^, & Neil B. Metcalfe^2^

^1^School of Life and Environmental Sciences, University of Sydney, Sydney, NSW, Australia

^2^Institute of Biodiversity, Animal Health and Comparative Medicine, University of Glasgow, Glasgow, UK

^3^School of Biological Sciences, Monash University, Melbourne, Victoria, Australia

*Corresponding author: [amanda.pettersen@sydney.edu.au](mailto:amanda.pettersen@sydney.edu.au)

**Literature search and data preparation**

To investigate the scaling relationship between offspring metabolic rate and size, we used data from Pettersen et al., (2019) (n = 68 papers) and a literature search in Web of Science (access gained from The University of Sydney on 26/11/2022). The search terms Metaboli* AND scaling AND (offspring OR larv* OR egg OR embryo) yielded 988 results that were then screened by LS and AKP for relevance using Rayyan software (Ouzzani et al. 2016). After reading abstracts, we reduced the list of papers to 145. After reading the full paper, our list was further reduced to 67. The primary reasons for exclusion were if studies did not measure offspring metabolic rate (n = 449), or the study organism was not an ectotherm (n = 464). We chose to include papers found within the final list that were not found in our Web of Science search but were relevant to our review (n = 59). Measures of metabolic rate were converted into ml O_2_ h^-1^ and offspring mass converted to mg (wet mass). We converted measures of dry mass to wet mass assuming 70% water content (Maino et al. 2017), where wet mass = dry mass * (1/0.3). Prior to analysis offspring mass and metabolic rate were log_10_ transformed. Data and references included are presented in **Table S1**.

**Statistical analysis**

A phylogeny was produced for 191 ectotherm species for which metabolic rate data was available, using Open Tree of Life (OpenTreeofLife et al. 2019) (**Figure S1**). All analyses were conducted in R version 4.0.3 (R Core Team 2020) and the R package *rotl* (Michonneau et al. 2016) was used to synthesise the phylogenetic tree, to control for autocorrelation created by the shared evolutionary history of species in our dataset. We used phylogenetic comparative analysis in a Bayesian framework to quantify the relationship between offspring metabolic rate and mass. We fit multi-response generalised linear mixed models using the R package *MCMCglmm* (Hadfield 2010). We used Deviance Information Criterion (DIC) values for model comparison and selection (Spiegelhalter et al. 2002), and results for the different models provided in **Table S2**. We first fit a simple animal model for Log_10_ Metabolic Rate with no fixed effects (m1), and with the random effect of ‘animal’ (i.e., species). We then added fixed effects of Log_10_ Offspring wet mass (m2), Temperature (average temperature for each species-level measurement) (m3), and the interaction of Log_10_ Offspring wet mass and Temperature (m4). For the offspring strategy comparison, we fit two separate models: m4.Feeding and m4.Nonfeeding for feeding and non-feeding offspring, respectively. For random effect priors, we used an inverse gamma prior, e.g., for m1 specifying *V* = 1 and *nu* = 0.002 (where *V* specifies variance and *nu* represents the degree of belief in *V*). Given that most species were represented by individual studies (191 species across 180 papers), we were unable to account for within- verses among-study effects. We used a sampling scheme of 65,000 iterations, with a thinning interval of 50, and a burn-in of 50,000 providing at least 1000 samples from the posterior distribution. Autocorrelation was low (lag values < 0.1) and trace plots showed well mixing chains for all parameters.

**Table S1. Offspring mass (converted to mg) and offspring metabolic rate (in ml O_2_ h^-1^) data extracted for 191 ectotherm species.** Mean environmental temperature for each species was estimated based on the mean temperature measured for each study. Offspring type refers to whether non-feeding eggs, embryos, or larvae (NF), compared with feeding larvae (F) were measured.

| Species | Offspring feeding type | Mean temperature (°C) | Mean mass (mg) | Mean metabolic rate (ml O_2_ h^-1^) | Source |
| --- | --- | --- | --- | --- | --- |
| Amphibians | | | | | |
| Agalychnis callidryas | NF | 27.00 | 871.111 | 0.153047 | (Bouchard et al. 2016) |
| Ambystoma maculatum | NF | 25.00 | 11.529 | 0.002212 | (Wills 1936) |
| Anaxyrus terrestris | F | 27.00 | 46.000 | 0.062814 | (Noland and Ultsch 1981) |
| Chiromantis xerampelina | NF | 25.00 | 1.500 | 0.000893 | (Seymour and Loveridge 1994) |
| Crinia georgiana | NF | 18.5 | 2.733 | 0.000529 | (Seymour and Roberts 1995) |
| Crinia nimbus | NF | 10.00 | 38.700 | 0.000420 | (Mitchell and Seymour 2000) |
| Ensatina eschscholtzii | NF | 14.00 | 308.000 | 0.017905 | (Bradford 1984) |
| Geocrina vitellina | NF | 15.00 | 15.250 | 0.001509 | (Mitchell 2001) |
| Hyla chrysoscelis | F | 21.63 | 244.000 | 0.016415 | (Rowe et al. 2018) |
| Lithobates catesbeianus | F | 25.11 | 3969.000 | 0.498140 | (Rowe et al. 2018) |
| Lithobates sphenocephalus | F | 21.54 | 457.000 | 0.061521 | (Rowe et al. 2018) |
| Pseudis paradoxus | F | 25.00 | 13108.240 | 0.038663 | (de Souza and Kuribara 2006) |
| Pseudophryne bibronii | NF | 17.0 | 4.389 | 0.001164 | (Seymour et al. 1991) |
| Rana pipiens | F | 26.33 | 702.500 | 0.173569 | (Noland and Ultsch 1981) |
| Rhinella marinus | NF | 27.50 | 134.056 | 0.046892 | (van Uitregt et al. 2016) |
| Scaphiopus holbrookii | F | 21.63 | 155.000 | 0.018634 | (Rowe et al. 2018) |
| Triturus rivularis | NF | 20.00 | 18.261 | 0.002371 | (Connon 1947) |
| Triturus torosus | NF | 22.50 | 7.565 | 0.001324 | (Wills 1936) |
| Fishes | | | | | |
| Abramis ballerus | F | 20.00 | 192.436 | 0.142428 | (Keckeis and Schiemer 1992) |
| Achirus lineatus | F | 28.00 | 0.1559 | 0.000380 | (Houde and Schekter 1983) |
| Albarnus albarnus | F | 20.00 | 78.754 | 0.045600 | (Keckeis and Schiemer 1992) |
| Alburnus alburnus | F | 20.00 | 57.345 | 0.029118 | (Keckeis and Schiemer 1990) |
| Alosa pseudoharengus | NF | 19.50 | 6.162 | 0.005724 | (Klumb et al. 2003) |
| Anabas testudineus | F | 28.00 | 736.065 | 0.089987 | (Mishra and Singh 1979) |
| Anchoa mitchilli | NF | 26.00 | 0.2665 | 0.000588 | (Houde and Schekter 1983) |
| Archosargus rhomboidalis | NF | 26.00 | 0.120 | 0.000230 | (Houde and Schekter 1983) |
| Chalcalburnus chalcoides | F | 18.33 | 1320.864 | 0.572358 | (Kaufmann 1990) |
| Channa punctatus | F | 28.00 | 179.033 | 0.352524 | (Singh et al. 1982) |
| Chanos chanos | NF | 27.50 | 0.3858 | 0.000455 | (Walsh et al. 1991) |
| Chondrostoma nasus | NF | 14.50 | 20.350 | 0.000393 | (Kamler et al. 1998) |
| Clupea harengus | F | 10.27 | 1.253 | 0.001002 | (Almatar 1984) |
| Clupea harengus pallasi | NF | 12.85 | 0.346 | 0.001851 | (Eldridge et al. 1977) |
| Coregonus clupeaformis | NF | 5.00 | 9.500 | 0.000030 | (Mueller et al. 2015) |
| Coregonus lavaretus | F | 9.50 | 74.614 | 0.025133 | (Hanel and and Wieser 1996) |
| Cyclopterus lumpus | F | 6.50 | 123.158 | 0.021635 | (Killen et al. 2007) |
| Cynoscion nebulosus | F | 28.50 | 28.356 | 0.027882 | (Wuenschel et al. 2004) |
| Cyprinus carpio | F | 25.00 | 426.743 | 0.117980 | (Oikawa and Itazawa 1995) |
| Danio rerio | NF | 26.91 | 0.047 | 0.042220 | (Pettersen et al. 2019) |
| Dicentrarchus labrax | F | 20.00 | 1110.000 | 3.137837 | (Dalla Via et al. 1998) |
| Esox lucius | F | 15.00 | 372.151 | 0.115029 | (Wieser et al. 1992) |
| Gadus morhua | F | 7.55 | 239.207 | 0.018220 | (Laurence 1978) |
| Girardinichthys multiradiatus | F | 20.00 | 115.000 | 0.031500 | (Garduño Paz et al. 2020) |
| Girella laevifrons | F | 17.50 | 11330.000 | 1.582159 | (Pulgar et al. 2017) |
| Gymnodraco acuticeps | NF | -0.75 | 13.117 | 0.000090 | (Evans et al. 2006) |
| Heterodontus portusjacksoni | NF | 20.00 | 155500.000 | 0.537600 | (Rodda 2000) |
| Heteropneustes fossilis | F | 28.00 | 1329.471 | 0.135426 | (Sheel and Singh 1981) |
| Hippoglossus hippoglossus | F | 6.35 | 7.895 | 0.000365 | (Finn and Ronnestad 1995) |
| Hypomesus pretiosus | F | 14.60 | 0.518 | 0.163557 | (Garreton 1983) |
| Leuciscus cephalus | F | 18.40 | 795.733 | 0.371728 | (Wieser and Forstner 1986) |
| Leuresthes tenuis | F | 19.83 | 0.923 | 0.693333 | (Ehrlich and Muszynski 1982) |
| Limanda limanda | F | 10.00 | 2393.275 | 0.664659 | (Edwards et al. 1969) |
| Melanogrammus aeglefinus | F | 6.78 | 3.4396 | 0.004035 | (Laurence 1978) |
| Merluccius productus | F | 11.73 | 0.127 | 0.000350 | Bailey 1982 |
| Micropterus salmoides | NF | 19.00 | 0.9573 | 0.000039 | (Laurence 1969) |
| Mugil cephalus | NF | 26.5 | 0.110 | 0.000383 | (Walsh et al. 1989) |
| Myoxocephalus scorpius | F | 3.00 | 130.536 | 0.018264 | (Killen et al. 2007) |
| Neoceratodus forsteri | NF | 20.00 | 47.720 | 0.000402 | (Mueller et al. 2011) |
| Oncorhynchus mykiss | NF | 10.50 | 142.000 | 0.003733 | (Rombough 1994) |
| Oncorhynchus tshawytscha | NF | 8.70 | 340.500 | 0.006335 | (Rombough 1994) |
| Oreochromis niloticus | NF | 30.00 | 3.908 | 0.005337 | (De Silva et al. 1986) |
| Pagrus major | F | 20.00 | 0.987 | 0.000885 | (Oikawa and Itazawa 1992) |
| Paralichthys olivaceus | F | 18.00 | 87.730 | 0.025920 | (Yagi and Oikawa 2014) |
| Perca flavescens | F | 22.50 | 13.786 | 57.249691 | (Czesny et al. 2005) |
| Petromyzon marinus | F | 12.00 | 1485.809 | 0.045902 | (Tessier et al. 2018) |
| Pleuronectes platessa | F | 11.80 | 537.542 | 0.081445 | (Almatar 1984) |
| Pseudopleuronectes americanus | F | 6.67 | 0.066 | 0.002238 | (Laurence 1975) |
| Rachycentron canadum | F | 27.40 | 148.796 | 0.249775 | (Watson et al. 2010) |
| Rutilus rutilus | F | 18.38 | 110.897 | 0.045809 | (Wieser and Forstner 1986) |
| Salmo gairdneri | NF | 9.67 | 777.770 | 0.077321 | (Rombough 1988) |
| Salmo salar | NF | 6.75 | 127.100 | 0.000351 | (Robertsen et al. 2014) |
| Salmo trutta fario | NF | 7.07 | 17.860 | 0.006474 | (Wood 1932) |
| Salvelinus alpinus | NF | 5.00 | 38.710 | 0.004281 | (Gruber and Wieser 1983) |
| Salvelinus fontinalis | NF | 7.00 | 98.000 | 0.002700 | (Cook et al. 2018) |
| Scardinius erythrophthalmus | F | 18.40 | 620.920 | 0.248326 | (Wieser and Forstner 1986) |
| Scomber japonicus | NF | 20.00 | 0.133 | 0.000350 | (Hunter and Kimbrell 1980) |
| Scomber scombrus | NF | 16.00 | 1.778 | 0.041184 | (Giguère et al. 1988) |
| Siganus randalli | NF | 28.33 | 1.524 | 0.002898 | (Nelson and Wilkins 1994) |
| Takifugu rubripes | F | 20.00 | 196.354 | 0.083797 | (Yagi et al. 2010) |
| Tautoga onitis | NF | 19.00 | 10.0778 | 0.004407 | (Laurence 1973) |
| Thunnus thynnus | NF | 25.50 | 19.990 | 0.065971 | (Blanco et al. 2020) |
| Xyrauchen texanus | NF | 15.0 | 149.750 | 0.000672 | (Bozek et al. 1990; Bestgen 2008) |
| Insects | | | | | |
| Alima pacifica | F | 28.00 | 66.667 | 0.026900 | (Ikeda et al. 2011) |
| Arctopsyche grandis | NF | 15.00 | 98.000 | 0.008561 | (Lowe et al. 1999) |
| Artemia salina | F | 16.00 | 0.030 | 0.000124 | (Eliassen 1952) |
| Balanus balanoides | F | 16.00 | 0.001 | 0.000002 | (Zeuthen 1947) |
| Belgica antarctica | F | 4.00 | 9.751 | 0.000788 | (Teets et al. 2019) |
| Calanus hyperborcus | F | 4.60 | 1.047 | 0.000162 | (Conover and Corner 1968) |
| Brevicoryne brassicae | F | 24.3 | 0.134 | 1.777143 | (Lamb 1961; Pratt et al. 2008) |
| Callinectes sapidus | NF | 21.00 | 0.390 | 0.034289 | (O’Leary Amsler and George 1984) |
| Cancer irroratus | F | 5.00 | 0.726 | 0.000141 | (Sastry and McCarthy 1973) |
| Cancer productus | F | 18.17 | 0.027 | 0.000009 | (Belman and Childress 1973) |
| Carcinus maenas | F | 18.33 | 0.157 | 0.000246 | (Dawirs 1983) |
| Chironomus sp | NF | 10.00 | 3.333 | 0.860000 | (McFarlane and McLusky 1972) |
| Culex pipiens | F | 22.5 | 4.155 | 2.430167 | (Richards 1964; Rueda et al. 1990) |
| Cyclograpus lavauxi | NF | 15.00 | 0.015 | 0.000000 | (Taylor and Leelapiyanart 2001) |
| Drosophila melanogaster | NF | 22.50 | 0.010 | 0.001397 | (de Moed et al. 1998) |
| Emerita talpoida | F | 25.00 | 2.000 | 0.002000 | (Schatzlein and Costlow 1978) |
| Heterozius rotundifrons | NF | 15.00 | 0.343 | 0.000001 | (Taylor and Leelapiyanart 2001) |
| Homarus americanus | F | 20.00 | 14.600 | 0.009512 | (Capuzzo and Lancaster 1979) |
| Hyas coarctatus | F | 10.50 | 0.741 | 0.000237 | (Jacobi and Anger 1985) |
| Manduca sexta | NF | 25.00 | 1678.098 | 2.183471 | (Callier and Nijhout 2012) |
| Melanoplus differentialis | NF | 22.58 | 5.067 | 0.713497 | (Richards 1964) |
| Oncopeltus fasciatus | NF | 19.10 | 0.296 | 0.000055 | (Richards and Suanraksa 1962) |
| Ostrinia nubilalis | NF | 25.00 | 0.084 | 0.872200 | (Richards 1964) |
| Pagarus bernhardus | F | 18.00 | 1.962 | 0.000222 | (Dawirs 1984) |
| Pagurus criniticornis | F | 25.00 | 0.200 | 0.000090 | (Vernberg 1983) |
| Panulirus cygnus | F | 20.50 | 253.528 | 0.049933 | (Lemmens 1994) |
| Panulirus omatus | F | 28.00 | 6.3700 | 0.002880 | (Ikeda et al. 2011) |
| Parapsyche elsis | NF | 15.00 | 111.000 | 0.008407 | (Lowe et al. 1999) |
| Petrolisthes laevigatus | NF | 12.00 | 1.2564 | 0.000103 | (Leiva et al. 2018) |
| Polypedilum sp | NF | 10.00 | 2.333 | 0.683333 | (McFarlane and McLusky 1972) |
| Punulirus interruptus | F | 18.17 | 0.217 | 0.000080 | (Belman and Childress 1973) |
| Rhithropanopeus harrisii | F | 25.00 | 0.204 | 0.000237 | (Levine and Sulkin 1979) |
| Solenopsis invicta | NF | 25.00 | 3.006 | 0.000683 | (Vogt and Appel 1999) |
| Tribolium confusum | NF | 19.20 | 0.0513 | 0.020868 | (Richards 1964) |
| Uca pugilator | F | 15.00 | 0.015 | 0.000015 | (Vernberg and Gostlow 1966) |
| Uca pugnax | F | 15.00 | 0.015 | 0.000015 | (Vernberg and Gostlow 1966) |
| Uca rapax | F | 15.00 | 0.012 | 0.000009 | (Vernberg and Gostlow 1966) |
| Uca thayeri | F | 15.00 | 0.014 | 0.000011 | (Vernberg and Gostlow 1966) |
| Marine invertebrates | | | | | |
| Acropora tenuis | NF | 28.00 | 0.013 | 0.000086 | (Richmond 1988) |
| Arenicola marina | NF | 15.83 | 1553.580 | 0.064357 | (De Cubber et al. 2019) |
| Asterina miniata | NF | 15.00 | 0.005 | 0.000009 | (Hoegh-Guldberg and Manahan 1995) |
| Bugula neritina | NF | 17.83 | 0.015 | 0.020690 | (Pettersen et al. 2019) |
| Carcinus aestuarii | NF | 20.00 | 0.0579 | 0.000616 | (Bartolini et al. 2013) |
| Centrostephanus coronatus | F | 20.00 | 0.019 | 0.000019 | (Shilling 1995) |
| Chasmagnathus granulata | NF | 18.00 | 0.182 | 0.033202 | (Ismael et al. 1998) |
| Cherax quadricarinatus | NF | 26.5 | 43.000 | 0.001487 | (King 1994; Garcı́a-Guerrero et al. 2003) |
| Crassostrea gigas | NF | 21.67 | 0.003 | 0.000005 | (Hoegh-Guldberg and Manahan 1995) |
| Crassostrea virginica | NF | 20.50 | 0.020 | 0.000004 | (Fuchs et al. 2017) |
| Crepidula fornicata | F | 24.00 | 0.0154 | 0.000044 | (Dobberteen and Pechenik 1987) |
| Daphnia magna | NF | 21.00 | 0.087 | 0.000135 | (Andrewartha and Burggren 2012) |
| Dendraster excentricus | NF | 15.00 | 0.003 | 0.000009 | (Hoegh-Guldberg and Manahan 1995) |
| Doryteuthis opalescens | F | 14.00 | 2.350 | 0.000001 | (Vidal et al. 2002) |
| Euphuasia superba | NF | 0.33 | 128.000 | 7.142667 | (Quetin and Ross 1989) |
| Helix aspera | NF | 20.00 | 2831.523 | 0.363326 | (Czarnoleski et al. 2008) |
| Hyas araneus | NF | 10.62 | 0.739 | 0.111181 | (Jacobi and Anger 1985) |
| Jasus edwardsii | F | 18.00 | 1.472 | 0.000410 | (Bermudes et al. 2008) |
| Lithodes maja | F | 9.00 | 0.662 | 0.001408 | (Anger 1996) |
| Loligo opalescens | F | 15.00 | 0.022 | 0.000077 | (Hurley 1976) |
| Loligo reynaudii | NF | 16.25 | 2.010 | 0.000001 | (Martins et al. 2010) |
| Marseniopsis mollis | F | -1.00 | 0.047 | 0.000000 | (Peck et al. 2006) |
| Metopaulias depressus | F | 25.00 | 1345.975 | 0.714569 | (Anger and Schuh 1992) |
| Mytilus edulis | NF | 12.00 | 0.0003 | 0.000012 | (Sprung 1984) |
| Octopus maya | NF | 24.00 | 83.564 | 2.931643 | (Caamal-Monsreal et al. 2016) |
| Ostrea edulis | F | 21.00 | 0.001 | 0.000001 | (Crisp 1974) |
| Pagurus bernhardus | F | 15.00 | 0.911 | 0.000496 | (Anger et al. 1990) |
| Pocillopora damicornis | NF | 27.49 | 0.388 | 0.000092 | (Edmunds et al. 2011) |
| Sagmariasus verreauxi | NF | 22.36 | 73.366 | 0.008870 | (Fitzgibbon et al. 2014) |
| Seriatopora hystrix | NF | 26.70 | 0.104 | 0.000058 | (Edmunds et al. 2011) |
| Strongylocentrotus purpuratus | NF | 15.5 | 0.002 | 0.008848 | (Padilla-Gamiño et al. 2013; Tellis et al. 2013) |
| Stylophora pistillata | NF | 27.20 | 0.392 | 0.000322 | (Edmunds et al. 2011) |
| Taliepus dentatus | F | 15.00 | 0.379 | 0.173667 | (Storch et al. 2011) |
| Thais haemastoma canaliculata | F | 24.00 | 0.026 | 0.000067 | (Dobberteen and Pechenik 1987; Roller and Stickle 1989) |
| Torellia mirabilis | F | -1.00 | 0.059 | 0.000000 | (Peck et al. 2006) |
| Watersipora subtorquata | NF | 17.50 | 0.018 | 0.030646 | (Pettersen et al. 2015) |
| Reptiles | | | | | |
| Apalone spinifera | NF | 28.50 | 6700.000 | 0.252570 | (Ligon and Lovern 2012) |
| Aspis cerastes | NF | 30.00 | 3393.500 | 0.739000 | (Dmi’el 1970) |
| Bassiana duperreyi | NF | 27.50 | 356.450 | 0.070663 | (Booth et al. 2000) |
| Caretta caretta | NF | 29.80 | 30333.333 | 1.369396 | (Reid et al. 2009) |
| Chelonia mydas | NF | 28.00 | 49730.000 | 1.987601 | (Booth et al. 2000) |
| Chelydra serpentina | NF | 29.00 | 11240.000 | 0.815572 | (Miller 1992) |
| Crocodylus johnstoni | NF | 30.00 | 80850.000 | 5.874090 | (Whitehead and Seymour 1990) |
| Crocodylus porosus | NF | 30.00 | 119400.000 | 9.647520 | (Whitehead and Seymour 1990) |
| Echis colorata | NF | 30.00 | 3343.000 | 0.612000 | (Dmi’el 1970) |
| Lampropholis guichenoti | NF | 27.50 | 158.250 | 0.039325 | (Booth et al. 2000) |
| Macrochelys temminckii | NF | 28.50 | 11500.000 | 0.518368 | (Ligon and Lovern 2012) |
| Natrix tessellata | NF | 30.00 | 2641.500 | 0.796000 | (Dmi’el 1970) |
| Phyllodactylus marmoratus | NF | 25.00 | 628.000 | 0.040235 | (Thompson and Russell 1999) |
| Pseudemys nelsoni | NF | 30.00 | 2512.438 | 1.007167 | (Kam 1993) |
| Python molurus bivittatus | NF | 31.00 | 242837.884 | 7.420160 | (Black et al. 1984) |
| Spalerosophis cliffordi | NF | 30.00 | 8264.500 | 1.472000 | (Dmi’el 1970) |
| Sphenodon punctatus | NF | 20.00 | 4110.000 | 0.123140 | (Booth and Thompson 1991) |
| Trachemys scripta | NF | 28.50 | 26100.000 | 0.326011 | (Ligon and Lovern 2012) |
| Trionyx triunguis | NF | 30.00 | 15730.000 | 1.496171 | (Leshem et al. 1991) |
| Vipera xanthina palaestinae | NF | 30.00 | 5537.000 | 0.809000 | (Dmi’el 1970) |

**Table S2. Summary of phylogenetic mixed effects models used to quantify the scaling relationship between offspring metabolic rate and mass for 191 ectotherm species.** CI refers to credible intervals for the scaling exponent, *b*, and *pMCMC* is the probability that *b* < 0. DIC (Deviance Information Criterion) was used for model comparison.

| **Model** | **Parameters** | **Estimate of *b***  **(Offspring mass exponent)** | **95% CI** | ***pMCMC*** | **DIC** |
| --- | --- | --- | --- | --- | --- |
| m1 | Phylogeny | - | - | - | 1236.453 |
| m2 | Mass +  Phylogeny | 0.690 | 0.585, 0.783 | <0.001 | 600.361 |
| m3 | Mass +  Temperature + Phylogeny | 0.659 | 0.557, 0.764 | <0.001 | 596.042 |
| m4 | Mass*Temperature + Phylogeny | 0.630 | 0.335, 0.972 | <0.001 | 598.014 |
| m3.Embryonic | Mass +  Temperature + Phylogeny | 0.623 | 0.487,  0.756 | <0.001 | - |
| m3.Larval | Mass +  Temperature + Phylogeny | 0.721 | 0.596,  0.843 | <0.001 | - |
| m3.Feeding | Mass +  Temperature + Phylogeny | 0.820 | 0.697, 0.959 | <0.001 | - |
| m3.Nonfeeding | Mass +  Temperature + Phylogeny | 0.598 | 0.466, 0.726 | <0.001 | - |

**References**

Almatar SM. 1984. Effects of acute changes in temperature and salinity on the oxygen uptake of larvae of herring (Clupea harengus) and plaice (Pleuronectes platessa). Mar Biol 80:117–24.

Andrewartha S, Burggren W. 2012. Transgenerational Variation in Metabolism and Life-History Traits Induced by Maternal Hypoxia in Daphnia magna. Physiol Biochem Zool 85:625–34.

Anger K. 1996. Physiological and biochemical changes during lecithotrophic larval development and early juvenile growth in the northern stone crab, Lithodes maja (Decapoda: Anomura). Marine Biology 126:283–96.

Anger K, Montú M, de Bakker C. 1990. Energy partitioning during larval development of the hermit crab Pagurus bernhardus reared in the laboratory. Journal of Experimental Marine Biology and Ecology 141:119–29.

Anger K, Schuh M. 1992. Bioenergetics of abbreviated larval development in the bromeliad crab, Metopaulias depressus (Decapoda: Grapsidae). Comparative Biochemistry and Physiology Part A: Physiology 103:507–18.

Bartolini F, Barausse A, Portner H, Giomi F. 2013. Climate change reduces offspring fitness in littoral spawners: a study integrating organismic response and long-term time-series. Glob Change Biol 19:373–86.

Belman BW, Childress JJ. 1973. Oxygen consumption of the larvae of the lobster Panulirus interruptus (Randall) and the crab Cancer productus Randall. Comp Biochem Physiol A Comp Physiol 44:821–28.

Bermudes M, Ritar AJ, Carter CG. 2008. The ontogeny of physiological response to light intensity in early stage spiny lobster (Jasus edwardsii) larvae. Comparative Biochemistry and Physiology Part A: Molecular & Integrative Physiology 150:40–45.

Bestgen KR. 2008. Effects of Water Temperature on Growth of Razorback Sucker Larvae. Western North American Naturalist 68:15–20.

Black CP, Birchard GF, Schuett GW, Black VD. 1984. Influence of incubation water content on oxygen uptake in embryos of the Burmese python (Python molurus bioittatus). In: Seymour RS, editor. Respiration and metabolism of embryonic vertebrates: Satellite Symposium of the 29th International Congress of Physiological Sciences, Sydney, Australia, 1983. Perspectives in vertebrate science Dordrecht: Springer Netherlands. p. 137–45.

Blanco E, Reglero P, Ortega A, Folkvord A, de la Gandara F, de Rojas A, Moyano M. 2020. First estimates of metabolic rate in Atlantic bluefin tuna larvae. J Fish Biol 97:1296–1305.

Booth DT, Thompson MB. 1991. A comparison of reptilian eggs with those of megapode birds. Egg incubation: its effects on embryonic development in birds and reptiles Cambridge University Press, Cambridge 325–44.

Booth DT, Thompson MB, Herring S. 2000. How incubation temperature influences the physiology and growth of embryonic lizards. J Comp Physiol B 170:269–76.

Bouchard S, O’Leary C, Wargelin L, Charbonnier J, Warkentin K, Bouchard SS, O’Leary CJ, Wargelin LJ, Charbonnier JF, Warkentin KM. 2016. Post-metamorphic carry-over effects of larval digestive plasticity. Funct Ecol 30:379–88.

Bozek MA, Paulson LJ, Wilde GR. 1990. Effects of ambient Lake Mohave temperatures on development, oxygen consumption, and hatching success of the razorback sucker. Environ Biol Fish 27:255–63.

Bradford DF. 1984. Physiological features of embryonic development in terrestrially-breeding plethodontid salamanders. In: Seymour RS, editor. Respiration and metabolism of embryonic vertebrates: Satellite Symposium of the 29th International Congress of Physiological Sciences, Sydney, Australia, 1983. Perspectives in vertebrate science Dordrecht: Springer Netherlands. p. 87–98.

Caamal-Monsreal C, Uriarte I, Farias A, Díaz F, Sánchez A, Re D, Rosas C. 2016. Effects of temperature on embryo development and metabolism of O. maya. Aquaculture 451:156–62.

Callier V, Nijhout H. 2012. Supply-Side Constraints Are Insufficient to Explain the Ontogenetic Scaling of Metabolic Rate in the Tobacco Hornworm, Manduca sexta. PLoS One 7.

Capuzzo JM, Lancaster BA. 1979. Some physiological and biochemical considerations of larval development in the American lobster, Homarus Americanus Milne Edwards. Journal of Experimental Marine Biology and Ecology 40:53–62.

Connon FE. 1947. A comparative study of the respiration of normal and hybrid Triturus embryos and larvae. Journal of Experimental Zoology 105:1–24.

Conover RJ, Corner EDS. 1968. Respiration and nitrogen excretion by some marine zooplankton in relation to their life cycles. Journal of the Marine Biological Association of the United Kingdom 48:49–75.

Cook CJ, Burness G, Wilson CC. 2018. Metabolic rates of embryos and alevin from a cold-adapted salmonid differ with temperature, population and family of origin: implications for coping with climate change. Conservation Physiology 6.

Crisp DJ. 1974. Energy relations of marine invertebrate larvae. Thalassia Jugoslavica 10:103–20.

Czarnoleski M, Kozlowski J, Dumiot G, Bonnet J, Mallard J, Dupont-Nivet M. 2008. Scaling of metabolism in Helix aspersa snails: changes through ontogeny and response to selection for increased size. J Exp Biol 211:391–99.

Czesny S, Graeb B, Dettmers J. 2005. Ecological consequences of swim bladder noninflation for larval yellow perch. Trans Am Fish Soc 134:1011–20.

Dalla Via J, Villani P, Gasteiger E, Niederstätter H. 1998. Oxygen consumption in sea bass fingerling Dicentrarchus labrax exposed to acute salinity and temperature changes: metabolic basis for maximum stocking density estimations. Aquaculture 169:303–13.

Dawirs RR. 1983. Respiration, energy balance and development during growth and starvation of Carcinus maenas L. larvae (Decapoda:Portunidae). Journal of Experimental Marine Biology and Ecology 69:105–28.

Dawirs RR. 1984. Respiratory metabolism of Pagurus bernhardus (Decapoda: Paguridae) megalopa. Mar Biol 83:219–23.

De Cubber L, Lefebvre S, Lancelot T, Denis L, Gaudron S. 2019. Annelid polychaetes experience metabolic acceleration as other Lophotrochozoans: Inferences on the life cycle of Arenicola marina with a Dynamic Energy Budget model. Ecol Model 411.

de Moed GH, de Jong G, Scharloo W. 1998. The energetics of growth in Drosophila melanogaster: effect of temperature and food conditions. Netherlands Journal of Zoology 48:169–88.

De Silva CD, Premawansa S, Keembiyahetty CN. 1986. Oxygen consumption in Oreochromis niloticus (L.) in relation to development, salinity, temperature and time of day. Journal of Fish Biology 29:267–77.

de Souza S, Kuribara C. 2006. Metabolic scaling associated with unusual size changes during larval development of the frog, Pseudis paradoxus. J Exp Biol 209:1651–61.

Dmi’el R. 1970. Growth and metabolism in snake embryos. J Embryol Exp Morphol 23:761–72.

Dobberteen RA, Pechenik JA. 1987. Comparison of larval bioenergetics of two marine gastropods with widely differing lengths of planktonic life, Thais haemastoma canal&data (Gray) and Crepidula fornicata (L.). Journal of Marine Biology and Ecology 19.

Edmunds P, Cumbo V, Fan T. 2011. Effects of temperature on the respiration of brooded larvae from tropical reef corals. J Exp Biol 214:2783–90.

Edwards RRC, Finlayson DM, Steele JH. 1969. The ecology of 0-group plaice and common dabs in Loch Ewe. II. Experimental studies of metabolism. Journal of Experimental Marine Biology and Ecology 3:1–17.

Ehrlich KF, Muszynski G. 1982. Effects of temperature on interactions of physiological and behavioural capacities of larval California grunion: Adaptations to the planktonic environment. Journal of Experimental Marine Biology and Ecology 60:223–44.

Eldridge MB, Echeverria T, Whipple JA. 1977. Energetics of Pacific Herring (Clupea harengus pallasi) Embryos and Larvae Exposed to Low Conentrations of Benzene, a Monoaromatic Component of Crude Oil. Transactions of the American Fisheries Society 106:452–61.

Eliassen E. 1952. The energy-metabolism of Artemia salina in relation to body size, seasonal rhythms, and different salinities. Univ Bergen, Norway, Arbok, Nturvit R 11:1–17.

Evans C, Pace L, Cziko P, Marsh A, Cheng C, DeVries A. 2006. Metabolic energy utilization during development of Antarctic naked dragonfish (Gymnodraco acuticeps). Polar Biol 29:519–25.

Finn R, Ronnestad I. 1995. Respiration, nitrogen and energy-metabolism of developing yolk sac larvae of Atlantic Halibut (Hippoglossus hippoglossus L). Comp Biochem Physiol A-Mol Integr Physiol 111:647–71.

Fitzgibbon Q, Jeffs A, Battaglene S. 2014. The Achilles heel for spiny lobsters: the energetics of the non-feeding post-larval stage. Fish Fish 15:312–26.

Fuchs H, Specht J, Adams D, Christman A. 2017. Turbulence induces metabolically costly behaviors and inhibits food capture in oyster larvae, causing net energy loss. J Exp Biol 220:3419–31.

Garcı́a-Guerrero M, Villarreal H, Racotta IS. 2003. Effect of temperature on lipids, proteins, and carbohydrates levels during development from egg extrusion to juvenile stage of Cherax quadricarinatus (Decapoda: Parastacidae). Comparative Biochemistry and Physiology Part A: Molecular & Integrative Physiology 135:147–54.

Garduño Paz MV, Méndez Sánchez JF, Burggren W, García Martínez JLA. 2020. Metabolic rate and hypoxia tolerance in Girardinichthys multiradiatus (Pisces: Goodeidae), an endemic fish at high altitude in tropical Mexico. Comparative Biochemistry and Physiology Part A: Molecular & Integrative Physiology 239:110576.

Garreton MS. 1983. Bioenergetics of yolk utilisation in embryos and yolk-sac in larvae of the surf-melt Hypomesijs pretiosus pretiosus (Girard, 1855) under different incubation temperatures.

Giguère L, Coté B, St-Pierre J-J. 1988. Metabolic rates scale isometrically in larval fishes. Mar Ecol Prog Ser 50:13–19.

Gruber K, Wieser W. 1983. Energeties of Development of the Alpine Chart, Salvelinus Mpinus, in Relation to Temperature and Oxygen. 9.

Hadfield JD. 2010. MCMC Methods for Multi-Response Generalized Linear Mixed Models: The MCMCglmm R Package. Journal of Statistical Software 33:1–22.

Hanel R, and Wieser W. 1996. Growth of swimming muscles and its metabolic cost in larvae of whitefish at different temperatures. Journal of Fish Biology 48:937–51.

Hoegh-Guldberg O, Manahan D. 1995. Coulometric measurement of oxygen-consumption during development of marine invertebrate embryos and larvae. J Exp Biol 198:19–30.

Houde ED, Schekter RC. 1983. Oxygen uptake and comparative energetics among eggs and larvae of three subtropical marine fishes. Marine Biology 72:283–93.

Hunter JohnR, Kimbrell CarolA. 1980. Early life history of Pacific mackerel, Scomber japonicus. 13.

Hurley AC. 1976. Feeding, behavior, food consumption, growth, and respiration of the squid Loligo Opalescens raised in the laboratory. Fishery Bulletin 74:176–82.

Ikeda T, Smith G, McKinnon AD, Hall M. 2011. Metabolism and chemical composition of phyllosoma larvae, with special reference to the tropical rock lobster Panulirus ornatus (Decapoda; Palinuridae). Journal of Experimental Marine Biology and Ecology 405:80–86.

Ismael D, Anger K, Moreira GS. 1998. Influence of temperature on larval survival, development, and respiration in Chasmagnathus granulata (Crustacea, Decapoda). Helgoländer Meeresunters 51:463–75.

Jacobi C, Anger K. 1985. Effect of temperature on respiration of larval stages of Hyas araneus and H. coarctatus (Decapoda, Majidae). Mar Ecol Prog Ser 26:181–86.

Kam Y-C. 1993. Physiological effects of hypoxia on metabolism and growth of turtle embryos. Respiration Physiology 92:127–38.

Kamler E, Keckei H, Bauer-Nemeschkal E. 1998. Temperature-induced changes of survival, development and yolk partitioning in Chondrostoma nasus. Journal of Fish Biology 53:658–82.

Kaufmann R. 1990. Respiratory Cost of Swimming in Larval and Juvenile Cyprinids. Journal of Experimental Biology 150:343–66.

Keckeis H, Schiemer F. 1990. Consumption, growth and respiration of bleak, Alburnus alburnus (L.), and roach, Rutilus rutilus (L.), during early ontogeny. Journal of Fish Biology 36:841–51.

Keckeis H, Schiemer F. 1992. Food consumption and growth of larvae and juveniles of three cyprinid species at different food levels. Environ Biol Fish 33:33–45.

Killen SS, Costa I, Brown JA, Gamperl AK. 2007. Little left in the tank: metabolic scaling in marine teleosts and its implications for aerobic scope. Proc R Soc B 274:431–38.

King CR. 1994. Growth and survival of redclaw crayfish hatchlings (Cherax quadricarinatus von Martens) in relation to temperature, with comments on the relative suitabitity of Cherax quadricarinatus and Cherax destructor for culture in Queensland. Aquaculture 122:75–80.

Klumb R, Rudstam L, Mills E. 2003. Comparison of alewife young-of-the-year and adult respiration and swimming speed bioenergetics model parameters: Implications of extrapolation. Trans Am Fish Soc 132:1089–1103.

Lamb KP. 1961. Some Effects of Fluctuating Temperatures on Metabolism, Development, and Rate of Population Growth in the Cabbage Aphid, Brevicoryne Brassicae. Ecology 42:740–45.

Laurence GC. 1969. The Energy Expenditure of Largemouth Bass Larvae, Micropterus salmoides, during Yolk Absorption. Transactions of the American Fisheries Society 98:398–405.

Laurence GC. 1973. Influence of Temperature on Energy Utilization of Embryonic and Prolarval Tautog, *Tautoga onitis*. J Fish Res Bd Can 30:435–42.

Laurence GC. 1975. Laboratory growth and metabolism of the winter flounder Pseudopleuronectes americanus from hatching through metamorphosis at three temperatures. Mar Biol 32:223–29.

Laurence GC. 1978. Comparative growth, respiration and delayed feeding abilities of larval cod (Gadus morhua) and Haddock (Melanogrammus aeglefinus) as influenced by temperature during laboratory studies. Mar Biol 50:1–7.

Leiva F, Garces C, Verberk W, Care M, Paschke K, Gebauer P. 2018. Differences in the respiratory response to temperature and hypoxia across four life-stages of the intertidal porcelain crab Petrolisthes laevigatus. Mar Biol 165.

Lemmens JWTJ. 1994. The Western Rock lobster Panulirus cygnus (George, 1962) (Decapoda: Palinuridae): the effect of temperature and developmental stage on energy requirements of pueruli. Journal of Experimental Marine Biology and Ecology 180:221–34.

Leshem A, Ar A, Ackerman RA. 1991. Growth, Water, and Energy Metabolism of the Soft-Shelled Turtle (*Trionyx triunguis*) Embryo: Effects of Temperature. Physiological Zoology 64:568–94.

Levine DM, Sulkin SD. 1979. Partitioning and utilization of energy during the larval development of the xanthid crab, Rhithropanopeus harrisii (Gould). Journal of Experimental Marine Biology and Ecology 40:247–57.

Ligon DB, Lovern MB. 2012. Interspecific Variation in Temperature Effects on Embryonic Metabolism and Development in Turtles. ISRN Zoology 2012:1–13.

Lowe W, Hauer F, Lowe W, Hauer F. 1999. Ecology of two large, net-spinning caddisfly species in a mountain stream: distribution, abundance, and metabolic response to a thermal gradient. Can J Zool-Rev Can Zool 77:1637–44.

Maino JL, Pirtle EI, Kearney MR. 2017. The effect of egg size on hatch time and metabolic rate: theoretical and empirical insights on developing insect embryos. Functional Ecology 31:227–34.

Martins RS, Roberts MJ, Vidal ÉAG, Moloney CL. 2010. Effects of temperature on yolk utilization by chokka squid (Loligo reynaudii d’Orbigny, 1839) paralarvae. Journal of Experimental Marine Biology and Ecology 386:19–26.

McFarlane A, McLusky DS. 1972. The oxygen consumption of chironomid larvae from loch leven in relation to temperature. Comparative Biochemistry and Physiology Part A: Physiology 43:991–1001.

Michonneau F, Brown JW, Winter DJ. 2016. rotl: an R package to interact with the Open Tree of Life data. Methods in Ecology and Evolution 7:1476–81.

Miller JC. 1992. Temperature-Dependent Development of the Convergent Lady Beetle (Coleoptera: Coccinellidae). Environmental Entomology 21:197–201.

Mishra AP, Singh BR. 1979. Oxygen uptake through water during early life of Anabas testudineus (Bloch). Hydrobiologia 66:129–33.

Mitchell NJ. 2001. The energetics of endotrophic development in the frog Geocrinia vitellina (Anura: Myobatrachinae). Physiol Biochem Zool 74:832–42.

Mitchell NJ, Seymour RS. 2000. Effects of Temperature on Energy Cost and Timing of Embryonic and Larval Development of the Terrestrially Breeding Moss Frog, *Bryobatrachus nimbus*. Physiological and Biochemical Zoology 73:829–40.

Mueller CA, Eme J, Manzon RG, Somers CM, Boreham DR, Wilson JY. 2015. Embryonic critical windows: changes in incubation temperature alter survival, hatchling phenotype, and cost of development in lake whitefish (Coregonus clupeaformis). J Comp Physiol B 185:315–31.

Mueller CA, Joss JMP, Seymour RS. 2011. The energy cost of embryonic development in fishes and amphibians, with emphasis on new data from the Australian lungfish, Neoceratodus forsteri. J Comp Physiol B 181:43–52.

Nelson S, Wilkins S. 1994. Growth and respiration of embryos and larvae of the rabbitfish Siganus-randalli (Pisces, Siganidae). J Fish Biol 44:513–25.

Noland R, Ultsch GR. 1981. The Roles of Temperature and Dissolved Oxygen in Microhabitat Selection by the Tadpoles of a Frog (Rana pipiens) and a Toad (Bufo terrestris). Copeia 1981:645–52.

Oikawa S, Itazawa Y. 1992. Relationship between metabolic-rate in vitro and body-mass in a marine teleost, Porgy pagrus-major. Fish Physiol Biochem 10:177–82.

Oikawa S, Itazawa Y. 1995. Comparison of Oxygen Consumption of Larval and Juvenile Carp Determined by Three Different Methods. Fisheries science 61:487–90.

O’Leary Amsler M, George RY. 1984. The effect of temperature on the oxygen consumption and developmental rate of the embryos of Callinectessapidus Rathbun. Journal of Experimental Marine Biology and Ecology 82:221–29.

OpenTreeofLife, Cranston KA, Redelings B, Reyes LLS, Allman J, McTavish EJ, Holder MT. 2019. Open Tree of Life Taxonomy. .

Ouzzani M, Hammady H, Fedorowicz Z, Elmagarmid A. 2016. Rayyan — a web and mobile app for systematic reviews, Systematic Reviews.

Padilla-Gamiño JL, Kelly MW, Evans TG, Hofmann GE. 2013. Temperature and CO _2_ additively regulate physiology, morphology and genomic responses of larval sea urchins, *Strongylocentrotus purpuratus*. Proc R Soc B 280:20130155.

Peck LS, Clarke A, Chapman AL. 2006. Metabolism and development of pelagic larvae of Antarctic gastropods with mixed reproductive strategies. Marine Ecology Progress Series 318:213–20.

Pettersen AK, White CR, Bryson-Richardson RJ, Marshall DJ. 2019. Linking life-history theory and metabolic theory explains the offspring size-temperature relationship. Ecology Letters 22:518–26.

Pettersen AK, White CR, Marshall DJ. 2015. Why does offspring size affect performance? Integrating metabolic scaling with life-history theory. Proceedings of the Royal Society B: Biological Sciences 282:20151946.

Pratt C, Pope TW, Powell G, Rossiter JT. 2008. Accumulation of Glucosinolates by the Cabbage Aphid Brevicoryne brassicae as a Defense Against Two Coccinellid Species. J Chem Ecol 34:323–29.

Pulgar J, Waldisperg M, Galban-Malagon C, Maturana D, Pulgar VM, Aldana M. 2017. UV radiation impacts body weight, oxygen consumption, and shelter selection in the intertidal vertebrate Girella laevifrons. Sci Total Environ 578:317–22.

Quetin LB, Ross RM. 1989. Effects of oxygen, temperature and age on the metabolic rate of the embryos and early larval stages of the Antarctic krill Euphausia superba Dana. Journal of Experimental Marine Biology and Ecology 125:43–62.

R Core Team. 2020. R: A language and environment for statistical computing. R Foundation for Statistical Computing, Vienna, Austria.

Reid KA, Margaritoulis D, Speakman JR. 2009. Incubation temperature and energy expenditure during development in loggerhead sea turtle embryos. Journal of Experimental Marine Biology and Ecology 378:62–68.

Richards AG. 1964. The Generality of Temperature Effects on Developmental Rate and on Oxygen Consumption in Insect Eggs. Physiological Zoology 37:199–211.

Richards AG, Suanraksa S. 1962. Energy Expenditure During Embryonic Development Under Constant Versus Variable Temperatures (oncopeltus Fasciatus (dallas))1. Entomologia Experimentalis et Applicata 5:167–78.

Richmond R. 1988. Competency and dispersal potential of planula larvae of a spawning versus a brooding coral. Proc 6th Int Coral Reef Symp 2:827–31.

Robertsen G, Armstrong J, Nislow K, Herfindal I, McKelvey S, Einum S, Robertsen G, Armstrong JD, Nislow KH, Herfindal I, McKelvey S, Einum S. 2014. Spatial variation in the relationship between performance and metabolic rate in wild juvenile Atlantic salmon. J Anim Ecol 83:791–99.

Rodda KR (Kate R. 2000. Development in the Port Jackson shark embryo / by Kate R. Rodda. (Thesis).

Roller RA, Stickle WB. 1989. Temperature and salinity effects on the intracapsular development, metabolic rates, and survival to hatching of Thais haemastoma canaliculata (Gray) (Prosobranchia:Muricidae) under laboratory conditions. Journal of Experimental Marine Biology and Ecology 125:235–51.

Rombough PJ. 1988. Growth, aerobic metabolism, and dissolved oxygen requirements of embryos and alevins of steelhead, *Salmo gairdneri*. Can J Zool 66:651–60.

Rombough PJ. 1994. Energy Partitioning During Fish Development: Additive or Compensatory Allocation of Energy to Support Growth? Functional Ecology 8:178.

Rowe C, Crandall E, Rowe CL, Crandall EA. 2018. The acute thermal respiratory response is unique among species in a guild of larval anuran amphibians-Implications for energy economy in a warmer future. Sci Total Environ 618:229–35.

Rueda LM, Patel KJ, Axtell RC, Stinner RE. 1990. Temperature-Dependent Development and Survival Rates of Culex quinquefasciatus and Aedes aegypti (Diptera: Culicidae). Journal of Medical Entomology 27:892–98.

Sastry AN, McCarthy JF. 1973. Diversity in metabolic adaptation of pelagic larval stages of two sympatric species of brachyuran crabs. Netherlands Journal of Sea Research 7:434–46.

Schatzlein FC, Costlow JD. 1978. Oxygen consumption of the larvae of the decapod crustaceans, Emerita talpoida (say) and Libinia emarginata leach. Comparative Biochemistry and Physiology Part A: Physiology 61:441–50.

Seymour RS, Geiser F, work(s): DFBR. 1991. Metabolic Cost of Development in Terrestrial Frog Eggs (Pseudophryne bibronii). Physiological Zoology 64:688–96.

Seymour RS, Loveridge JP. 1994. Embryonic and larval respiration in the arboreal foam nests of the African frog Chiromantis xerampelina. J Exp Biol 197:31–46.

Seymour RS, Roberts JD. 1995. Oxygen Uptake by the Aquatic Eggs of the Australian Frog *Crinia georgiana*. Physiological Zoology 68:206–22.

Sheel M, Singh BR. 1981. O2 uptake through water during early life of Heteropneustes fossilis (Bloch). Hydrobiologia 78:81–86.

Shilling F. 1995. Morphological and physiological responses of echinoderm larvae to nutritive signals. Am Zool 35:399–414.

Singh RP, Prasad MS, Mishra AP, Singh BR. 1982. Oxygen uptake through water during early life in Channa punctatus (Bloch) (Pisces; Ophicephaliformes). Hydrobiologia 87:211.

Spiegelhalter DJ, Best NG, Carlin BP, Van Der Linde A. 2002. Bayesian measures of model complexity and fit. Journal of the Royal Statistical Society: Series B (Statistical Methodology) 64:583–639.

Sprung M. 1984. Physiological energetics of mussel larvae (Mytilus edulis). I. Shell growth and biomass. Mar Ecol Prog Ser 17:283–93.

Storch D, Fernandez M, Navarrete S, Portner H. 2011. Thermal tolerance of larval stages of the Chilean kelp crab Taliepus dentatus. Mar Ecol-Prog Ser 429:157–67.

Taylor H, Leelapiyanart N. 2001. Oxygen uptake by embryos and ovigerous females of two intertidal crabs, Heterozius rotundifrons (Belliidae) and Cyclograpsus lavauxi (Grapsidae): scaling and the metabolic costs of reproduction. J Exp Biol 204:1083–97.

Teets N, Kawarasaki Y, Potts L, Philip B, Gantz J, Denlinger D, Lee R. 2019. Rapid cold hardening protects against sublethal freezing injury in an Antarctic insect. J Exp Biol 222.

Tellis MS, Lauer MM, Nadella S, Bianchini A, Wood CM. 2013. Ionic status, calcium uptake, and Ca2+-ATPase activity during early development in the purple sea urchin (Strongylocentrotus purpuratus). Comparative Biochemistry and Physiology Part A: Molecular & Integrative Physiology 166:272–77.

Tessier L, Long T, Wilkie M. 2018. Influence of body size, metabolic rate and life history stage on the uptake and excretion of the lampricide 3-trifluoromethyl-4-nitrophenol (TFM) by invasive sea lampreys (Petromyzon marinus). Aquat Toxicol 194:27–36.

Thompson M, Russell K. 1999. Growth and energetics of embryos of the gecko, Phyllodactylus marmoratus, a species with hard-shelled eggs. Herpetolog J 9:37–42.

van Uitregt V, Alton L, Heiniger J, Wilson R. 2016. Warmer temperatures reduce the costs of inducible defences in the marine toad, Rhinella marinus. J Comp Physiol B-Biochem Syst Environ Physiol 186:123–30.

Vernberg FJ. 1983. Environmental Adaptations Elsevier.

Vernberg FJ, Gostlow JD. 1966. Studies on the Physiological Variation between Tropical and Temperate-Zone Fiddler Crabs of the Genus Uca. IV. Oxygen Consumption of Larvae and Young Crabs Reared in the Laboratory. Physiological Zoology 39:36–52.

Vidal EAG, DiMarco FP, Wormuth JH, Lee PG. 2002. Influence of temperature and food availability on survival, growth and yolk utilization in hatchling squid. Bulletin of Marine Science 71:915–31.

Vogt J, Appel A. 1999. Standard metabolic rate of the fire ant, Solenopsis invicta Buren: effects of temperature, mass, and caste. J Insect Physiol 45:655–66.

Walsh WA, Swanson C, Lee C-S. 1991. Effects of development, temperature and salinity on metabolism in eggs and yolk-sac larvae of milkfish, Chanos chanos (Forsskål). Journal of Fish Biology 39:115–25.

Walsh WA, Swanson C, Lee C-S, Banno JE, Eda H. 1989. Oxygen consumption by eggs and larvae of striped mullet, Mugil cephalus, in relation to development, salinity and temperature. Journal of Fish Biology 35:347–58.

Watson A, Holt G, Watson AM, Holt GJ. 2010. Energy Budget of Early Juvenile Cobia, Rachycentron canadum. J World Aquacult Soc 41:224–34.

Whitehead PJ, Seymour RS. 1990. Patterns of Metabolic Rate in Embryonic Crocodilians *Crocodylus johnstoni* and *Crocodylus porosus*. Physiological Zoology 63:334–52.

Wieser W, Forstner H. 1986. Effects of temperature and size on the routine rate of oxygen consumption and on the relative scope for activity in larval cyprinids. J Comp Physiol B 156:791–96.

Wieser W, Laich A, Medgyesy N. 1992. Energy allocation and yield and cost of growth in young Esox-luciusand Coregonus-lavaretus (teleostei) - influence of species, prey type and body size. J Exp Biol 169:165–79.

Wills IA. 1936. The respiratory rate of developing amphibia with special reference to sex differentiation. Journal of Experimental Zoology 73:481–510.

Wood AH. 1932. The effect of temperature on the growth and respiration of fish embryos. Journal of Experimental Biology 9:271–76.

Wuenschel M, Werner R, Hoss D, Wuenschel M, Werner R, Hoss D. 2004. Effect of body size, temperature, and salinity on the routine metabolism of larval and juvenile spotted seatrout. J Fish Biol 64:1088–1102.

Yagi M, Kanda T, Takeda T, Ishimatsu A, Oikawa S, Yagi M, Kanda T, Takeda T, Ishimatsu A, Oikawa S. 2010. Ontogenetic phase shifts in metabolism: links to development and anti-predator adaptation. Proc R Soc B-Biol Sci 277:2793–2801.

Yagi M, Oikawa S. 2014. Ontogenetic phase shifts in metabolism in a flounder Paralichthys olivaceus. Sci Rep 4:7135.

Zeuthen E. 1947. Body size and metabolic rate in the animal kingdom. With special regard to the marine micro-fauna.
